# Retinal imaging technologies in cerebral malaria: a systematic review

**DOI:** 10.1186/s12936-023-04566-7

**Published:** 2023-04-26

**Authors:** Kyle J. Wilson, Amit Dhalla, Yanda Meng, Zhanhan Tu, Yalin Zheng, Priscilla Mhango, Karl B. Seydel, Nicholas A. V. Beare

**Affiliations:** 1 Department of Eye & Vision Sciences, University of Liverpool, Liverpool, UK; 2 Malawi-Liverpool-Wellcome Trust, Blantyre, Malawi; 3 Department of Ophthalmology, Sheffield Teaching Hospitals, Sheffield, UK; 4 School of Psychology and Vision Sciences, College of Life Science, The University of Leicester Ulverscroft Eye Unit, Robert Kilpatrick Clinical Sciences Building, Leicester Royal Infirmary, Leicester, UK; 5 St. Paul’s Eye Unit, Royal Liverpool University Hospitals, Liverpool, UK; 6 Liverpool Centre for Cardiovascular Science, University of Liverpool and Liverpool Heart and Chest Hospital, Liverpool, UK; 7 Department of Ophthalmology, Kamuzu University of Health Sciences, Blantyre, Malawi; 8 College of Osteopathic Medicine, Michigan State University, East Lansing, MI USA; 9 Blantyre Malaria Project, Kamuzu University of Health Sciences, Blantyre, Malawi

**Keywords:** Cerebral malaria, Malarial retinopathy, Fundus photography, Fluorescein angiography, Optical coherence tomography

## Abstract

**Background:**

Cerebral malaria (CM) continues to present a major health challenge, particularly in sub-Saharan Africa. CM is associated with a characteristic malarial retinopathy (MR) with diagnostic and prognostic significance. Advances in retinal imaging have allowed researchers to better characterize the changes seen in MR and to make inferences about the pathophysiology of the disease. The study aimed to explore the role of retinal imaging in diagnosis and prognostication in CM; establish insights into pathophysiology of CM from retinal imaging; establish future research directions.

**Methods:**

The literature was systematically reviewed using the African Index Medicus, MEDLINE, Scopus and Web of Science databases. A total of 35 full texts were included in the final analysis. The descriptive nature of the included studies and heterogeneity precluded meta-analysis.

**Results:**

Available research clearly shows retinal imaging is useful both as a clinical tool for the assessment of CM and as a scientific instrument to aid the understanding of the condition. Modalities which can be performed at the bedside, such as fundus photography and optical coherence tomography, are best positioned to take advantage of artificial intelligence-assisted image analysis, unlocking the clinical potential of retinal imaging for real-time diagnosis in low-resource environments where extensively trained clinicians may be few in number, and for guiding adjunctive therapies as they develop.

**Conclusions:**

Further research into retinal imaging technologies in CM is justified. In particular, co-ordinated interdisciplinary work shows promise in unpicking the pathophysiology of a complex disease.

## Background

Malaria is a parasitic infection caused by *Plasmodium* species, which remains a significant cause of morbidity and mortality globally. In 2020, there were an estimated 627,000 deaths due to malaria, of which 96% occurred in the African region and approximately 80% in children aged 0–5 years [1]. Severe malaria is defined as parasitaemia accompanied by one or more of impaired consciousness, prostration, multiple seizures, acidosis, anaemia, renal impairment, jaundice, pulmonary oedema, abnormal bleeding, shock and/or hyperparasitaemia in the absence of any other attributable cause. Cerebral malaria (CM) is a serious neurological manifestation of severe malaria characterized by coma (defined as Glasgow Coma Score < 11 in adults or Blantyre Coma Score < 3 in children, lasting more than one hour post-ictal in the presence of seizure activity) and parasitaemia. Mortality due to CM approaches 100% without treatment, falling to 10–20% when prompt treatment is given [2].

Malaria has been shown to cause a specific retinopathy with diagnostic and prognostic significance in CM [3, 4]. It is characterized by the presence of retinal haemorrhages, with or without white centres, retinal whitening, which may occur in the macula or the periphery, and retinal vessel discolouration to orange or white. It may also be associated with papilloedema [5].

The pathology seen in the retina in MR parallels the pathology seen in the brain in CM. Sequestration of *Plasmodium falciparum*-parasitized red blood cells (pRBCs) in the retinal vasculature is always accompanied by sequestration in the cerebral vasculature [6, 7]. Papilloedema, by definition indicating raised intracranial pressure, is associated with death in CM [8].

Moreover, maintenance of a stable extracellular fluid environment is achieved through a highly selective blood-tissue barrier in both the retina and the brain. Blood–brain barrier (BBB) dysfunction is thought to contribute to brain swelling and death [9]. Blood-retinal barrier (BRB) breakdown can be demonstrated pathologically by the increased incidence of cystoid macular oedema on histopathological assessment of the retina in cases of fatal CM [7]. Intraretinal cystic spaces contain fibrinogen, which is entirely intravascular in the presence of a functioning BRB. As the only part of the central nervous system which can be directly visualized with non-invasive methods, the retina provides a unique opportunity to study the pathophysiology of CM in vivo.

Advances in retinal imaging technology have led to the development of imaging-based screening for retinal conditions, such as diabetic retinopathy, and have revolutionized the clinical management of retinal and chorioretinal conditions in recent years. This review seeks to establish the role of retinal imaging technologies in CM.

## Methods

### Aims

We aim to systematically review the literature on retinal imaging technologies in CM to: explore the potential benefits of retinal imaging in diagnosis and prognostication in CM; establish insights into the pathophysiology of CM from retinal imaging; inform future directions for research on retinal imaging technologies in CM.

### Evidence acquisition

A systematic review of the literature was performed in July 2022 using the African Index Medicus, MEDLINE, Scopus and Web of Science databases. Subject headings and free-text protocols were applied singularly before being combined with AND/OR operators. The complete search strategy for each of the databases has been highlighted in Appendix 1.

**Fig. 1:**
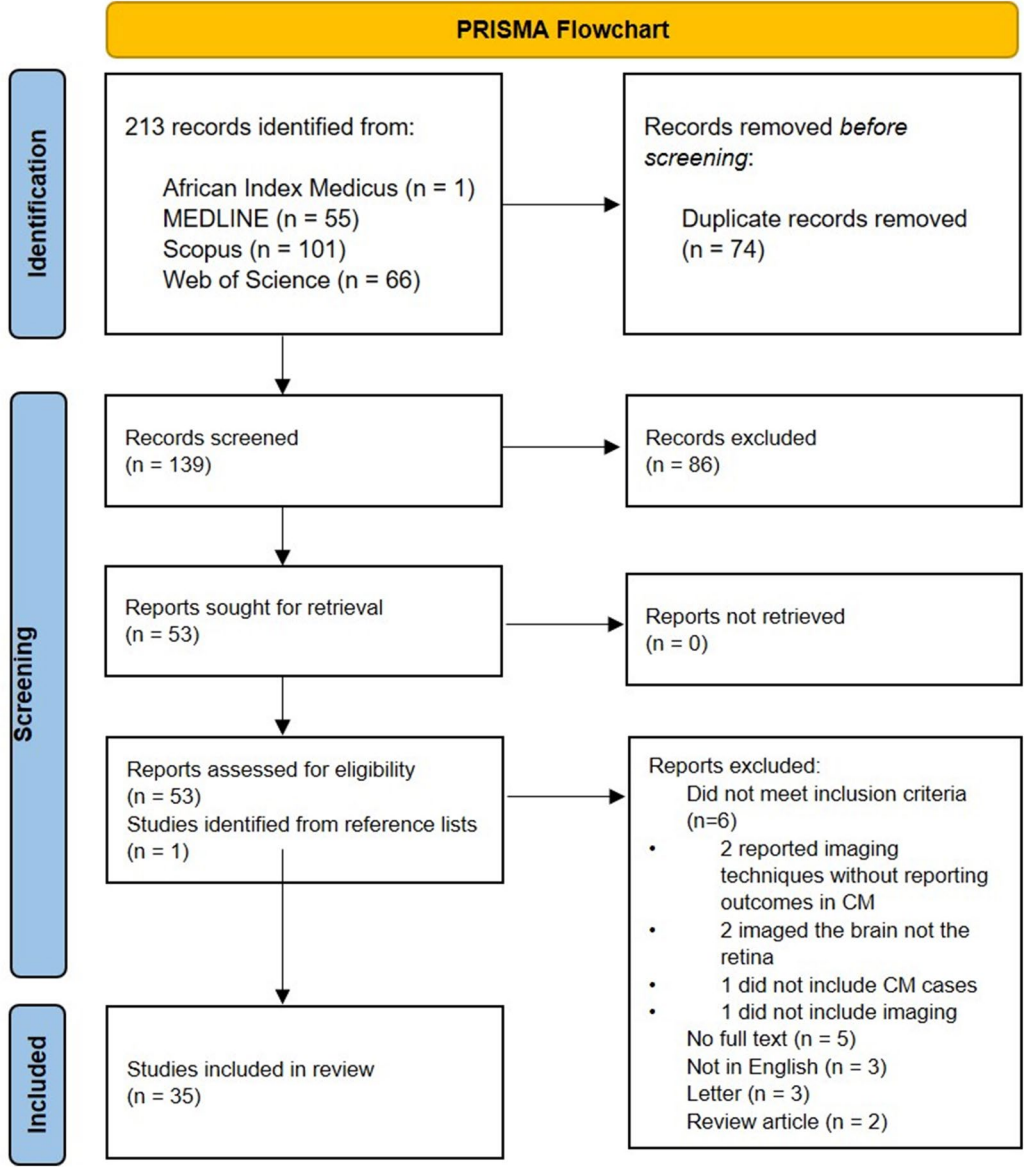
PRISMA flowchart

139 unique studies were identified from the database search. Abstracts were screened independently by two authors highlighting any studies or case reports that used retinal imaging technologies in cerebral malaria. Subsequent full-text screening of the included abstracts was performed by the same authors. All studies evaluating either a novel or established retinal imaging technique in human or experimental CM were included. Studies which included imaging but did not report imaging-related outcomes were excluded. Additionally, studies without experimental data on CM patients were excluded, along with reviews, case reports, letters and conference abstracts (without available full text). A third, independent author was consulted to resolve any disputes over inclusion and to verify the accuracy of data collection. Reference lists of included articles were manually searched for other relevant studies. A total of 35 full texts were included in the final analysis. Meta-analysis was precluded by heterogeneity in the literature. The PRISMA flowchart is shown in Fig. 1. Included papers are detailed in Appendix 2.

## Results

### Retinal imaging technologies in cerebral malaria

#### Retinal photography

Retinal photography allows non-ophthalmologists to acquire and grade fundus images, with well-documented practical applications in the assessment of retinopathy [10, 11]. Furthermore, where a problem is identified or there is uncertainty, the same images can be reviewed by an ophthalmologist or another suitably trained clinician without further inconveniencing the patient. This is particularly pertinent in low-resource environments, where there are relatively few ophthalmologists per capita, and patients often travel considerable distances to access health care. Given the established utility of MR in differentiating CM from coma of other cause, and that the effect of CM is felt primarily in the low- and middle-income countries (LMIC) of the African region, the potential advantages of retinal photography become even more clear [3, 12].

**Fig. 2:**
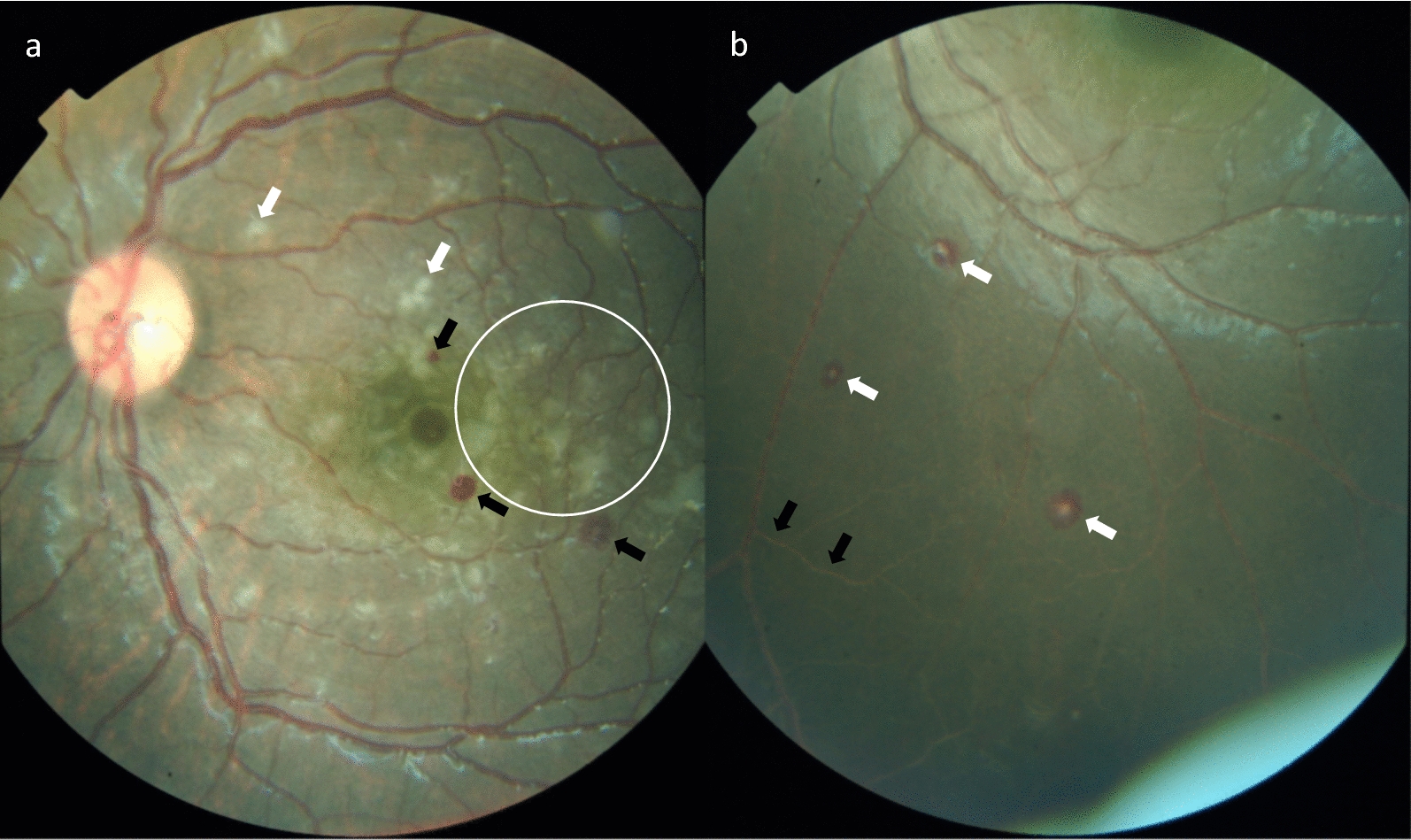
**a** Colour fundus photograph of the left eye of a child with cerebral malaria aged 28 months. Macular whitening is widespread (white circle and elsewhere) and there are retinal haemorrhages (black arrows). Cotton wool spots (white arrows) are whiter and more superficial than whitening. **b** Colour fundus photograph of the left eye of a different child with cerebral malaria aged 24 months. Extensive vessel discolouration (black arrows highlight one vessel segment) and white-centred haemorrhages (white arrows) are present

Clinicians have been aware of fundal changes in the retina of patients with severe malaria since the late nineteenth century [13]. Since then, many papers have described the characteristic changes, with several papers in the 80 s and 90 s systematically examining the retinae of patients with CM and correlating findings with outcome, some with photographic documentation [14–17]. Example images are presented in Fig. 2.

Despite this, only five studies which reported outcomes related to retinal photography were identified. Four reported photographic findings, one of which reported only minimal data on an unselected subgroup from a larger study, and one study sought to compare the effectiveness of different retinal cameras to binocular indirect ophthalmoscopy (BIO). Several studies evaluated artificial intelligence (AI) approaches to identifying MR using retinal photographic images. These will be addressed separately.

The four studies which reported their photographic findings are summarized in Table 1. The incidence of MR is roughly similar to that reported in clinical studies [4]. Interestingly, no adults had evidence of vessel discolouration, contrasting with the reported incidence of approximately 25% in African children [18]. It should be noted that vessel changes in MR are often very peripheral, and thus may be difficult to image using fundus photography.

**Table 1:** Retinal photographic findings in cerebral malaria

Study author	Year	Adult/Child	UM/SM/CM	No. patients	No. any MR (%)	No. haemorrhages (%)	No. whitening (%)		No. vessel discolouration (%)	No. papilloedema (%)
							Macular	Peripheral		
Sayeed	2011	Adult	Not stated	16	–	–	6 (38)	–	0 (0)	0 (0)
Maude	2009	Adult	CM	20	14 (70)	11 (55)	10 (50)	9 (45)	0 (0)	1 (5)
			SM	27	17 (63)	14 (52)	13 (48)	13 (48)	0 (0)	2 (7)
Hero^a^	1997	Child	SM (inc. CM)	40 (26)	31 (78)	25 (63)	15 (38)	23 (58)	5 (13)	5 (13)
Davis	1991	Adult	CM	5	4 (80)	2 (40)	–	–	–	–
			SM	13	5 (38)	0 (0)	–	–	–	–

*UM *uncomplicated malaria, *SM* severe malaria, *CM* cerebral malaria, – not recorded

^a ^Change in nomenclature of signs since publication in 1997 requires a degree of inference

Grading systems for MR have been developed at the University of Liverpool, with high levels of interobserver concordance in the hands of experienced ophthalmologists using BIO [5, 18, 19]. While BIO represents the gold standard for the detection of MR, it can be a difficult skill to learn, and grading requires both accurate examination and retention of much visual information while performing the examination. Grading of images from a retinal camera by ophthalmologists had an interobserver concordance of 100% in a study of Bangladeshi adults with severe malaria [20]. This is likely due to the static nature of camera images and the ability to digitally manipulate them with magnification and colour adjustment tools.

In a further study of Bangladeshi adults with MR, a subgroup was randomly selected to undergo retinal photography and this was graded and used as a control. Concordance between non-ophthalmologist observers was not reported, and the absence of an ophthalmologist’s assessment as the ‘gold standard’ test somewhat limits the utility of the data. However, the study did suggest that retinal photography was a more sensitive tool for identifying retinal whitening than ophthalmoscopy (direct and indirect) by a non-ophthalmologist [21].

A comprehensive study of vessel changes and retinal whitening including clinical, photographic, angiographic and pathological data found that orange vessel changes are strongly associated with death [22]. This study is evaluated in detail later.

Only Soliz et al*.* [23] directly compared the findings from retinal cameras to those of an ophthalmologist using BIO. Of the three cameras that they evaluated, the handheld Pictor Plus camera appeared to be superior, with sensitivity and specificity for identifying MR in patients with CM of 100% and 87%, respectively. Of note, all cameras tested failed to adequately image the peripheral retina; of importance given the previously mentioned predilection of vessel changes to occur in the periphery. Though the results are impressive and suggest a role for fundus photography in screening for MR, the study is limited by small numbers. The authors also comment that design shortcomings may limit utility in the field, specifically the absence of real-time feedback on image quality. In resource-limited environments users may be technicians without substantial ophthalmic training and therefore may not recognize that an image is of poor quality, potentially causing a delay to treatment if re-imaging is required.

#### Fundus fluorescein angiography (FA)

Fluorescein is an organic compound that, when stimulated with blue light (λ = 465–490 nm) emits a green light of longer wavelength (λ = 520–530 nm) [24]. FA was developed in the 1960s and permits detailed imaging of the retinal vasculature and real-time, in vivo visualization of vascular pathology. Fluorescein dye is injected intravenously before serial photographs are taken using a camera with a blue excitation filter and a green emission filter. Injected fluorescein remains entirely intravascular in uncompromised blood vessels but will leak if the BRB is at all compromised. Sequential imaging allows visualization of the retinal microvasculature in five phases: choroidal flush (pre-arterial), arterial, arteriovenous (laminar venous), venous and recirculation.

The similarities between the retinal and cerebral microcirculation mean that FA is a useful tool for understanding injury to the cerebral microvasculature in CM. Though it is possible to perform FA in low-resource environments with adapted equipment, it is not as straightforward as retinal photography and carries additional risks, such as anaphylaxis. Accordingly, its use as a screening tool is limited but it remains a useful tool to investigate pathophysiology.

Six studies which specifically addressed FA in CM were identified. These are summarized in Table 2. Studies which used AI approaches to interpreting FA images in CM are reported separately.

**Table 2:** Fundus fluorescein angiogram findings in cerebral malaria

Study author	Year	Adult/Child	UM/SM/CM	No. patients (No. CM^a^)	No. any abnormality (%)	No. CNP (%)			No. IVFDs (%)	No. FL (%)				
						Total	Macula	Periphery		LFL	PL	Vessel leak		Disc Leak
												Post-capillary venules	Larger Venules	
MacCormick^b^	2022	Child	CM	260	–	–	> 88%	> 57%	15% ^c^	18%	32%	> 29%	44%	-
Barrera^b^	2018	Child	CM	260	–	–	–	–	98% ^d^	–	–	–	-	-
MacCormick^b^	2015	Child	Not stated (MR +)	264 (259)	264 (100)	100%	100%	95%	60–90% ^d,e^	12% ^e^	30% ^e^	50% ^e^	60% ^e^	80% ^e^
Beare^b^	2008	Child	CM	34	28 (82)	26 (76)	19 (56)	22 (65)	9 (26)	15 (44)				
Hero	1997	Child	CM / SM	12 (11)	5 (42)	2 (17)	–	–	–	0 (0)	0 (0)	0 (0)		2 (17)
Davis	1992	Adult	CM	5	3 (60)	3 (60)	–	–	–	3 (60)				
			SM	13	4 (31)	3 (23)	–	–	–	2 (15)				

*UM* uncomplicated malaria, *SM* severe malaria, *CM* cerebral malaria, *CNP* capillary non-perfusion, *IVFD* intravascular filling defect, *LFL* large focal leak, *PL*, punctate leak, *MR*+ malarial retinopathy positive, – not recorded

^a ^where relevant

^b ^datasets may contain same patients

^c ^arteriolar

^d ^multiple vessels analysed, highest figures quoted (post capillary venules)

^e ^approximate figures

**Fig. 3:**
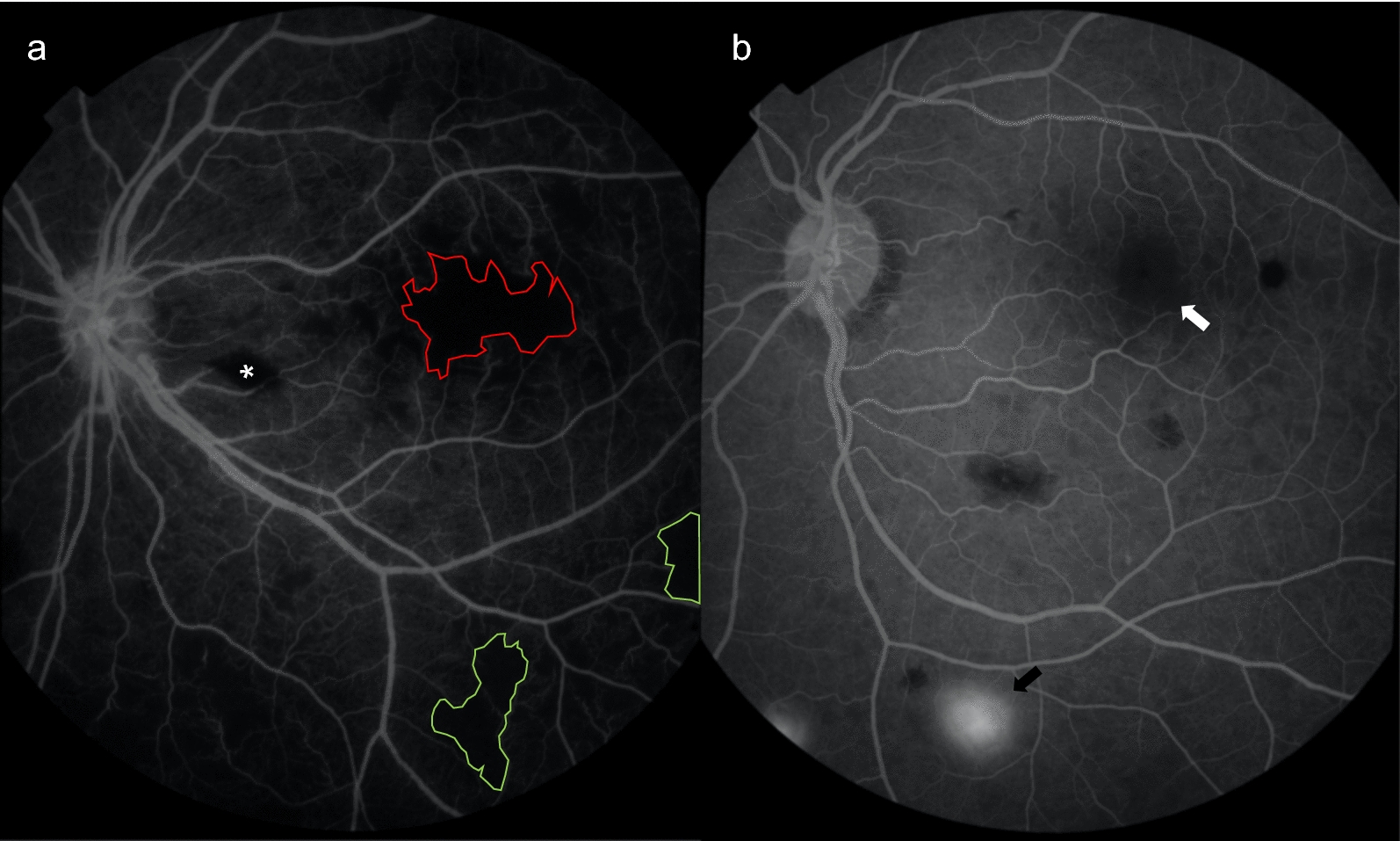
**a** Arteriovenous phase fluorescein angiogram of the left eye of a child with cerebral malaria aged 20 months shows macular (red outline) and peripheral (green outline) capillary non-perfusion. Note that haemorrhage (asterisk) masks fluorescence. **b** Venous phase angiogram of a different paediatric patient with cerebral malaria shows large focal leak (black arrow) and enlargement of the foveal avascular zone (white arrow), indicating capillary non-perfusion. Note again masking from haemorrhages

Early studies using film cameras by Davis et al. and Hero et al. used FA in a small number of patients. Both studies identified angiographic abnormalities including capillary non-perfusion (CNP), vessel leak and disc leak [8, 25]. Neither was sufficiently powered to accurately describe the range of changes seen or the prevalence of those changes. In addition, film cameras do not provide real-time feedback, so image quality may be compromised. Subsequent large studies using digital imaging have identified a range of angiographic findings which broadly can be grouped into CNP, leakage and intravascular filling defects (IVFD) [26, 27]. Examples are shown in Fig. 3.

CNP is caused when a capillary network fails to fill by the late arteriovenous phase. Images from earlier phases must not be used for grading, as the capillary network may not have had sufficient time to fill, thereby risking false positives. CNP is extremely common in CM, with some degree of macular CNP and peripheral CNP in 100% and 95% of gradable MR-positive cases, respectively, in one study [27]. It co-localizes to areas of retinal whitening, suggesting that whitening arises because of an ischaemic insult.

Fluorescein leak seen in CM can be grouped into one of three types: large focal leak (LFL), punctate leak (PL) and vessel leak (VL). Interestingly, LFL and PL appear to associate with brain swelling and death, whereas vessel leak and CNP appear to associate with neurological sequelae in survivors [28]. This may suggest that the rapid accumulation of retinal haemorrhages (seen as evolving LFLs on FA) indicates multiple focal breaks in the BRB, which is likely to be mirrored in the BBB and brain. This leakage of blood cells and large proteins is concurrent with a rapid fluid egress, a possible mechanism for brain swelling and death.

VL was histologically associated with perivascular fibrinogen on histopathological assessment in the same study [28]. It was also frequently related to areas of CNP and together VL and CNP were associated with neurological sequelae. The authors conclude that sequestration of pRBCs results in immunological dysregulation with BRB disruption and patchy ischaemia, resulting in vasogenic and cytotoxic oedema, a pathological process distinct from that seen in LFL. The association with neurological sequelae and not death suggests that, in the brain, this may result in significantly less fluid egress than LFL, thus rendering this BBB compromise more survivable, albeit with neurological injury resulting from ischaemia and reperfusion.

Barrera et al*.* [22] retrospectively analysed a large, prospectively collected dataset of Malawian children with CM, using clinical, photographic, angiographic and pathological analysis to establish whether sequestration of pRBCs is clinically visible in the retina and whether this correlates with outcome. IVFDs in the venous circulation were demonstrated in an overwhelming majority of cases (98.3%) on review of the angiographic images. Though much less frequent, there was an increased risk of death when IVFDs affected the arteriolar circulation. In addition, detailed pathological analysis and cross-referencing of angiographic imaging demonstrate that intravascular filling defects are areas of parasite sequestration. Where parasites are sequestered there are marked changes in the retinal neurovascular unit with endothelial cell dysfunction alongside pericyte dysfunction and loss. Changes in pericyte function were paralleled in the brain in an unpublished small sub-analysis by the same authors. These findings add further weight to the hypothesis that BBB breakdown secondary to parasite sequestration leads to neurological injury. The mechanisms underpinning which children will die and which survive remain unclear, but may be related to host-parasite interactions and the host immune response.

#### Optical coherence tomography (OCT)

**Fig. 4:**
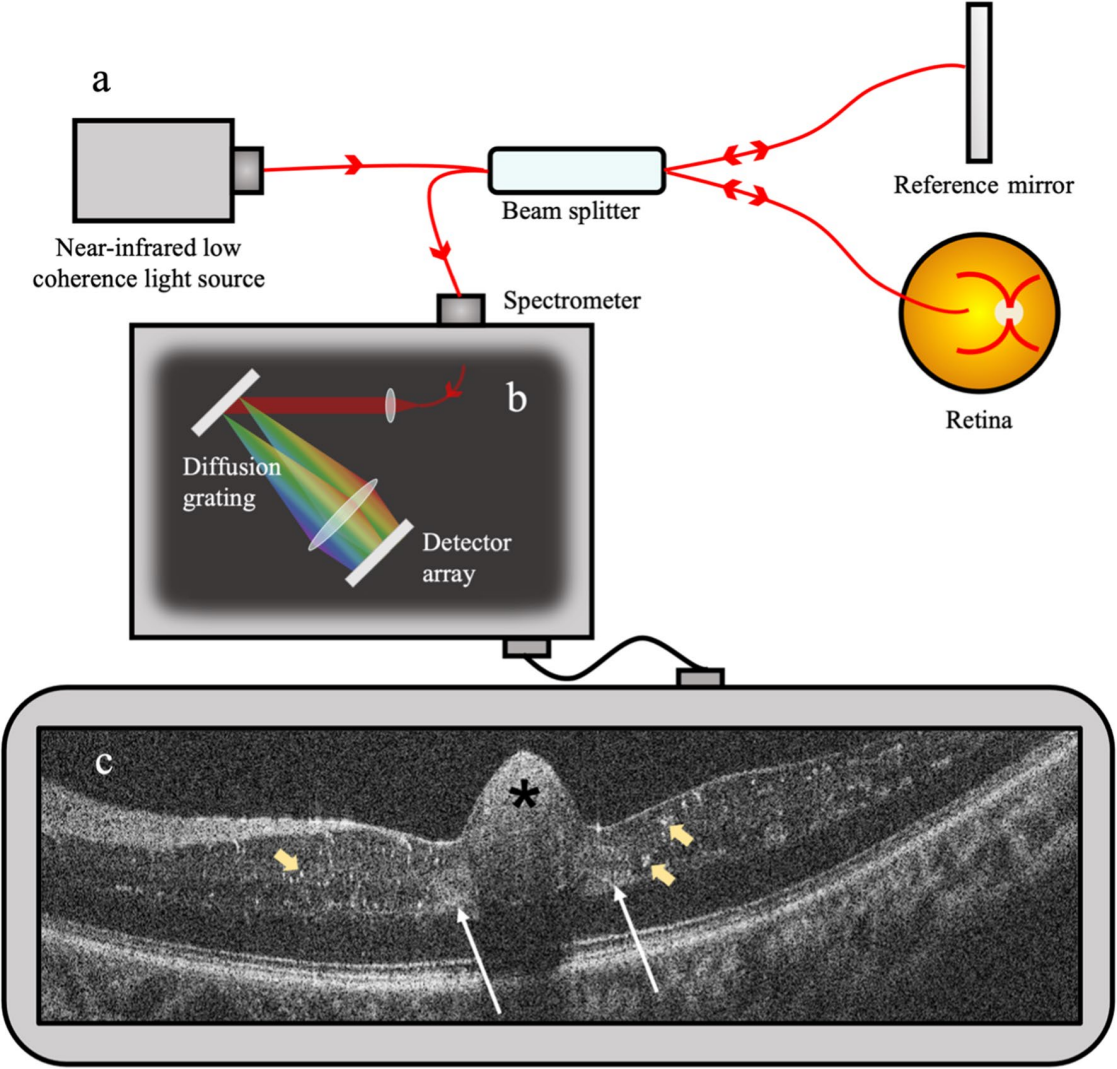
**a** SD-OCT machine: a low-coherence near-infrared beam is split such that it is directed at both the sample and a reference mirror. The beam is reflected from reflective surfaces (cellular interfaces) in the sample and from the reference mirror. **b** In the spectrometer, the recombined beams undergo Fourier transformation, and the resulting interference is detected by the detector array. Signals are processed to form OCT images. c) OCT scan of the left eye of a child aged 15 months. Hyperreflective capillaries (pale yellow arrows), hyperreflective areas (long white arrows) and haemorrhage (black asterisk) are visible

Optical coherence tomography (OCT) can be used to generate high-resolution cross-sectional images of the retina and optic nerve *in-vivo*. A beam of near-infrared light is split into a probe beam and reference beam, the former of which is reflected from the tissue which is being analysed and the latter from a mirror. The beams are recombined and the interference pattern is interpreted by an interferometer. Multiple data points over a 2 mm distance are integrated, producing an image analogous to an *in-vivo* histopathological section. A spectral-domain OCT (SD-OCT) is illustrated in Fig. 4. OCT facilitates the detection of subtle and sub-clinical macular oedema and other changes to retinal integrity, as well as helping to differentiate causes of optic nerve head swelling. Only one paper describing changes on OCT in human CM [29]. A further study uses the technology in a murine model of CM [30].

Tu et al*.* [29] describe several novel features on OCT (see Fig. 4). Firstly, they identified hyperreflective areas (HRAs) affecting the inner retinal layers which, when large, colocalize to areas of retinal whitening and CNP on fundus photography and FA, respectively. The outer layers are spared as their blood supply is derived from the choroid, in which there is little to no sequestration. A hyperreflective signal from the inner retinal layers is seen in retinal artery occlusion and gives support to the theory that hyperreflectivity of the inner retina results from cytotoxic oedema secondary to tissue hypoxia in CM.

Cotton wool spots were visible in the retinal nerve fibre layer (RNFL) as raised and well-defined lesions with lower signal density when compared to HRAs [29]. They were much more common on OCT than have previously been appreciated clinically (37 vs. 5%), suggesting clinical misclassification as retinal whitening- [18, 29]. Cotton wool spots are caused by axonal injury in the RNFL, often secondary to ischaemia. This has been demonstrated in the paediatric retina in CM by increased levels of β-amyloid precursor protein in the RNFL during histopathological assessment [7].

The presence of hyperreflective vessels and hyperreflective capillaries in the inner retinal layers is unique to CM. This provides another potential example of in vivo visualization of sequestered pRBCs [29]. However, it is not yet known how hyperreflectivity of vessels relates to visible vessel abnormalities and their histopathological correlates.

A further study assessing the retinal microvasculature in a murine model of CM used OCT in addition to confocal scanning laser ophthalmoscopy and histopathological analysis showed colocalization of regions of interest on OCT with parasite sequestration in the blood vessels, supporting the conclusions reached by Tu et al*.* [29] that vessel hyperreflectivity represents sequestered pRBCs [30].

OCT identified cystoid macular oedema in approximately 10% of patients, significantly less than has been observed on histopathology (53.1%) [7, 29]. Patients that die and go on to autopsy are more likely to have severe disease, which could account for this difference.

Finally, longitudinal analysis of OCTs from survivors showed that HRAs develop into areas of retinal thinning by one month, which is more pronounced at one year [29]. This may be reflected in the brain, where significant brain atrophy is seen in those with neurodevelopmental deficits on magnetic resonance imaging (MRI) at one month [31].

These OCT findings have not yet been correlated with clinical outcome. OCT shows promise as a bedside tool for the assessment of CM, as well as potentially aiding our understanding of the pathogenesis of the disease. However, high expense of OCT technologies, a lack of expertise and parts to repair damaged or faulty machines, and the steep learning curve for non-ophthalmic practitioners using handheld devices may limit scalability in LMICs.

#### Automated detection of retinal lesions

The approaches to automated image analysis can be loosely grouped into two major strategies: traditional computer vision and deep learning. Simply, traditional computer vision (TCV) uses algorithms based on functions such as colour thresholding and pixel counting to perform functions such as segmentation and detection of saliency (standing out against the background). It requires significant processing power but requires fewer lines of code than machine learning techniques. TCV can be computationally slow. AI in the form of deep learning is an important and growing area of retinal image analysis [32]. Traditional image analysis is time-consuming and costly, requiring trained graders to assess images and rigorous quality control processes. AI has the potential to reduce the need for human graders, freeing them up for quality control and assessing images which are equivocal or difficult to grade using algorithms. While the mathematics of AI is beyond the scope of this review, some key concepts are summarized below [33].

#### Deep learning

The rise of deep learning is based on the development of convolutional neural networks. A model is developed and trained using a bank of images, such that it can begin to recognize intrinsic features within an image without additional expert input and to make decisions based on those features. After training from a training dataset, deep learning models can be applied to a test dataset to further evaluate the models’ performance. Several approaches to deep learning have been trialled in MR detection.

#### Supervised vs Unsupervised vs Semi-Supervised Learning

Supervised learning uses a set of labelled training data to teach a model to recognize features of the same category’s data. A supervised model is usually more accurate than an unsupervised model, but the start-up cost is significant, requiring a large dataset and human intervention for labelling.

Unsupervised learning models cluster and analyse data on their own, to identify patterns within the data and thus summarize the patterns’ characteristics and then recognize them. The key difference between unsupervised and supervised learning is the use of labelled data. A semi-supervised or weakly-supervised model uses a mixture of labelled and unlabelled data to assist a model benefiting from both labelled and unlabelled information and features.

#### Transfer learning

Transfer learning utilizes a model pre-trained with a large amount of data similar to the data the new model will analyse and then fine-tunes it using a small amount of purpose-specific training data. This process significantly reduces the computational cost of training a model^34^. Transfer learning for detection of MR used a model trained to identify diabetic retinopathy and adapted it with a small MR training dataset [34].

Most papers on imaging in MR relate to AI analysis. This is likely because models can be developed on pre-existing datasets, without the need to recruit further patients. Additionally, many of the features of MR on colour fundus imaging and FA are present in other retinopathies, most notably diabetic retinopathy. Accordingly, there are researchers developing algorithms which can detect lesions in both MR and diabetic retinopathy. This generalizability increases the number of researchers interested and capable of tackling the issue, as well as increasing access to funding.

**Fig. 5:**
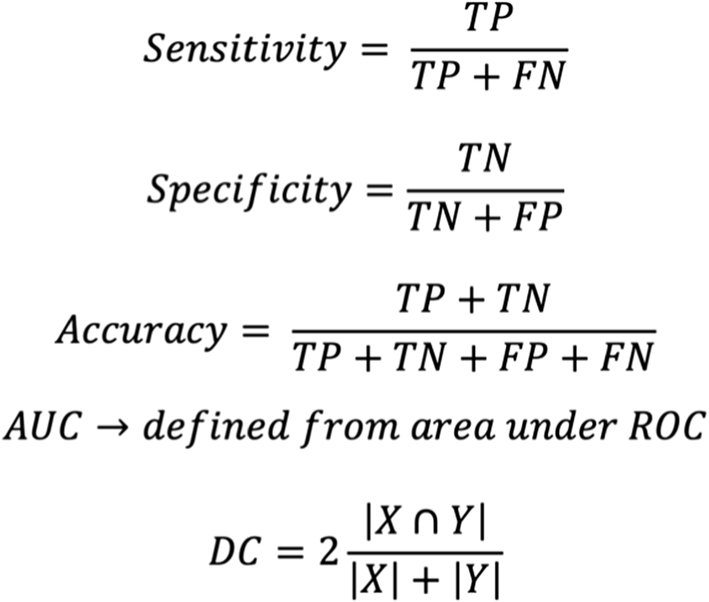
Statistical tests in image analysis may be applied at the pixel or region level. *TP* true positive; *TN* true negative; *FP* false positive; *FN* false negative; *AUC* area under the curve; *ROC* receiver operating curve, 1-specificty against sensitivity; *DC* dice coefficient, |X| number of elements in X, X ∩ Y elements that are similar in X and Y

21 papers which reported findings on the automated detection of retinal lesions on either colour fundus photography or FA were identified. The results of these tables are summarized in Table 3. Metrics used in the evaluation of AI models are summarized in Fig. 5. Dice coefficient (DC) is a metric that is frequently applied in image segmentation analysis to compare pixels or regions identified by a model with those identified by one or more experts. Because children without MR should be thoroughly worked-up for alternative causes of coma, most algorithms were optimized for high specificity, rather than sensitivity. In addition to sensitivity and specificity, the receiver operating curve (ROC) is also used, based on which area under curve (AUC) can be computed. AUC tends to be more informative than accuracy when the number of cases is imbalanced between different groups.

**Table 3:** Summary of automated detection of cerebral malaria from retinal imaging

Study Author	Year	Imaging modality	Method	Lesion detected	Sensitivity	Specificity	Accuracy	AUC	DC
Li	2022	FA	Weakly-supervised DL	LFL/PL	0.91 ± 0.02 ^a^	0.97 ± 0.02 ^a^	0.96 ± 0.02 ^a^	0.94 ± 0.02 ^a^	0.85 ± 0.02 ^a^
Kurup	2020	Colour	Transfer DL	Haemorrhage & Whitening	0.90	1	0.81	0.98	–
Chen	2020	Colour	Supervised DL	Haemorrhage	–	–	–	0.94	0.995
Yan	2019	FA	Superpixel-based TCV	LFL / PL	0.95	0.95	–	0.95	–
Zhao	2018	FA	Superpixel-based TCV	LFL / PL	0.95	0.95	–	0.95	–
Rochim	2018	FA	Saliency-based TCV	LFL / PL	-	0.9	0.98	–	–
Joshi	2018	Colour	TCV	Haemorrhage	0.79	0.93	–	0.88	–
				Whitening	0.67	0.77	–	0.75	–
				MR	0.68	0.92	–	0.82	–
Zhao	2017	FA	Saliency-based TCV	LFL / PL	0.93 ± 0.03	0.96 ± 0.02	0.91 ± 0.03	0.94 ± 0.02	0.82 ± 0.03
Zhao	2017	FA	Saliency-based TCV	LFL / PL	0.83 ± 0.08	0.83 ± 0.03	0.81 ± 0.04	0.83 ± 0.05	–
MacCormick	2017	FA	Spatial statistical model TCV	CNP	–	–	–	–	–
Joshi	2017	Colour	Unsupervised TCV	Haemorrhage	0.73	0.96	–	0.89	–
				Whitening	0.65	0.94	–	0.81	–
				Vessel Discolouration	0.62	1	–	0.85	–
				MR	0.95	1	–	0.97	–
Akram	2017	Colour	TCV	Whitening	0.90	0.92	–	0.9	–
Zhao	2015	FA	Saliency-based TCV	IVFD	0.75	0.74	0.74	0.74	–
Zhao	2015	FA	Saliency-based TCV	LFL	0.95	–	–	–	–
				PL	0.82	–	–	–	–
				Vessel Leak	0.81	0.82	0.8	0.84	–
Joshi	2015	Colour	TCV	Whitening	0.88 ^b^	0.65 ^b^	–	0.8	–
					0.82 ^c^	0.89 ^c^	–	–	–
Ashraf	2015	Colour	TCV	Whitening	–	–	0.92	–	–
Ashraf	2015	Colour	TCV	Haemorrhage	0.95	0.96	0.97	0.95	–
				Whitening	–	–	0.92	–	–
				CWS	0.82	–	–	–	–
Agurto	2015	Colour	TCV	Vessel Discolouration	0.94 ^b^	0.67 ^c^	0.85	0.87	–
					0.68 ^b^	1 ^c^	0.85	–	–
Zheng	2014	FA	Texture-based TCV	CNP	0.73 ± 0.14	0.91 ± 0.06	0.89 ± 0.04	–	–
Saleem	2014	Colour	TCV	Haemorrhage	0.95	0.96	0.97	0.95	–
Joshi	2012	Colour	Splat-based TCV	Haemorrhage	0.81	0.97	–	0.91	–

*AUC* area under the curve, *DC* dice coefficient, *DL* deep learning, *MR* malarial retinopathy, *TCV* traditional computer vision

^a^only LFL included in quantitative analysis

^b^tuned for sensitivity

^c^tuned for specificity

Image analysis is not independent of image acquisition and, while the results of AI analysis are extremely impressive, it should be noted that current camera technologies used in MR studies do not capture the periphery sufficiently well to accurately grade MR. Additionally, the algorithms with the best performance were often trained and tested using high-quality images captured with expensive cameras, some of which are table-mounted. Table-mounted cameras are not practical for capturing images in comatose children and the high expense of advanced cameras is a barrier to their use outside of the research environment in LMICs. While further efforts are made to increase the sensitivity and specificity of AI image analysis and reduce analysis time, parallel research should concentrate on the production of a low-cost widefield camera and development of software which facilitates accurate assembly of montage images from handheld cameras.

#### Other technologies

Beare et al*.* [35] hypothesized that outcome in CM could correlate with changes in optic nerve head blood flow and tested their hypothesis using laser Doppler flowmetry, which uses the principle of the Doppler effect in scattered laser light to quantify movement of erythrocytes [35]. They demonstrated an increased blood volume in children with papilloedema but did not show any correlation between blood-flow and outcome. The study was limited by the lack of a suitable control group or normative data in children.

Two papers used less common or novel imaging techniques to evaluate the retinas of mice infected with *Plasmodium berghei*. The mouse model of cerebral malaria is well-characterized and has been used extensively in research into the pathophysiological mechanisms underlying CM [36].

Hyperspectral imaging is an adaption of fundus photography that uses computerized analysis of spectral reflections from the retina to quantify blood oxygenation in the vasculature. In murine CM reduced oxygenation was observed with this technique, possibly indicating parasitic haematophagy [37]. Though this finding may illustrate an important process in malarial pathogenesis its relation to malarial retinopathy remains unclear, and the technique does not appear to have been used in human subjects with malaria yet.

Finally, laser speckle imaging has been used to demonstrate changes to blood flow in mouse retinas using camera-phone technology [38]. Laser speckle imaging detects blurring in the speckle pattern (noise) created by interference in light emitted from a coherent laser light source. As components of the sample move this interference pattern changes and an image can be generated. The technique is non-contact and non-invasive but does not provide absolute values for blood flow. Sequential imaging would be required to show changes in blood flow which may limit utility in deteriorating CM patients.

## Discussion

Ophthalmic imaging techniques have contributed to improved diagnosis and increased prognostic accuracy in CM and have furthered our understanding of the pathophysiology of this complex and life-threatening disease. Nevertheless, a complete understanding of the pathophysiology of CM remains elusive. Sequestration of pRBCs in brain microvasculature is the pathological hallmark of the disease and there is now considerable evidence that this process is visible in the retinal microvasculature with ophthalmic imaging and clinical examination [12, 22, 29].

Sequestration occurs in most microvasculature of the grey and white matter of affected brains and has been associated with BBB dysfunction, haemorrhage, thrombosis, and axonal damage [39]. This finding is supported by FA and OCT studies which show vascular sequestration in post-capillary venules of 98.3% of eyes (FA) and capillary hyper-reflectivity in 93% of eyes (OCT) [22, 29]. Cytoadherence of pRBCs to the vascular endothelium is principally mediated by the *P. falciparum* erythrocyte membrane protein 1 (PfEMP-1) encoded by parasite *var* genes. PfEMP-1 binds to several different host receptors including endothelial protein-C receptor (EPCR) and intercellular adhesion molecule 1 (ICAM-1) which are expressed on brain and retina microvascular endothelial cells. Recent work has demonstrated that differential expression of *var* gene transcripts accurately differentiates between CM and uncomplicated malaria [40]. Further, binding of pRBCs to EPCR limits the production of activated protein C, with resultant inflammation, fibrin production, clot formation and BBB/BRB dysfunction [41]. High levels of EPCR *var* transcripts have been implicated in severe brain swelling, which is common in fatal paediatric CM cases [42, 43]. After pRBCs are sequestered in the vessel walls, schizogony leads to cell rupture and results in the release of haemozoin and histidine-rich protein 2 (HRP-2). It is postulated that HRP-2 disrupts the tight junctions between endothelial cells, thus rendering the BBB ineffective [44]. Increased expression of EPCR *var* transcripts may lead to increased sequestration. Where there is intense focal sequestration and simultaneous schizont rupture, local inflammation, dysregulation of coagulation and increased HRP-2 release contribute to the development of focal breaches in the BBB/BRB. The increased disruption to the BBB/BRB is clinically visible as LFL on FA; seemingly evolving haemorrhage. Ultimately, multiple microhaemorrhages result in loss of blood cells and large proteins to the extravascular compartment, fluid shift, cerebral oedema and death. Further investigation of haemorrhage evolution in the retina could be useful, potentially providing a clinical biomarker for increased risk of brain swelling. This would be particularly useful if the assertion by MacCormick et al*.* [28] that neurological sequelae and death from CM arise because of two distinct pathophysiological processes proves true, given that they would each merit different approaches to treatment. A current trial assessing treatment protocols for brain swelling in CM is ongoing, but none have assessed limiting ischaemic injury [45].

Neurological problems are common in survivors of cerebral malaria [46]. These can include cognitive deficits, motor difficulties, memory problems and cortical visual impairment. Seizures are common and if untreated can contribute further to developmental delay [47]. CNP and vessel leak correlated with neurological sequelae in survivors of CM and HRAs on OCT co-localize to areas of CNP [28, 29]. Further, HRAs develop into areas of thinning by one month, which remain at one year. This may correlate with MRI scan at one month, which shows significant brain atrophy in survivors of CM [31]. Retinal imaging could be a useful tool in identifying those survivors at risk of neurological disability, which, if coordinated with further research into neuroprotective treatments in CM, could be of great advantage in the future.

## Conclusion

CM continues to have a significant impact in LMICs of the African region. Available research clearly shows retinal imaging is useful both as a clinical tool for the assessment of CM and as a scientific instrument to aid our understanding of the condition. Modalities which can be performed at the bedside, such as fundus photography and OCT, are best positioned to take advantage of AI image analysis, unlocking the clinical potential of retinal imaging for diagnosis, and guiding adjunctive therapies as they develop. This will require cross-cutting research work, coordinated between different disciplines. Further research into retinal imaging technologies in CM is both necessary and justified.

## Data Availability

All data generated or analysed during this study is included in the published article [and its supplementary information files].

## Appendix 1

### African Index Medicus Search Strategy

(tw:((tw:(optical coherence tomography)) OR (tw:(fluorescein angiography)) OR (tw:(indocyanine green)) OR (tw:(photography)) OR (tw:(retinal photography)) OR (tw:(retinal imaging)) OR (tw:(retinal camera)) OR (tw:(fundus photography)) OR (tw:(fundus imaging)) OR (tw:(fundus camera)) OR (tw:(optical imaging)))) AND (tw:(malaria)).

### Ovid MEDLINE Search Strategy

(exp Tomography, Optical Coherence/ OR exp Fluorescein Angiography/ OR exp Photography/ OR exp Indocyanine Green/ OR exp Optical Imaging/ OR fundus photography.mp. OR fundus imaging.mp. OR fundus camera.mp. OR fundus photograph.mp. OR retinal photography.mp. OR retinal imaging.mp. OR retinal camera.mp. OR retinal photograph.mp.) AND (exp Malaria, Cerebral/ OR (exp Retina/ AND exp Malaria/) OR malarial retinopathy.mp.)

### SCOPUS Search Strategy

( ( TITLE-ABS-KEY ( optical AND coherence AND tomography) OR TITLE-ABS-KEY ( fluorescein AND angiography) OR TITLE-ABS-KEY ( indocyanine AND green) OR TITLE-ABS-KEY ( photography) OR TITLE-ABS-KEY ( optical AND imaging) OR TITLE-ABS-KEY ( fundus AND camera) OR TITLE-ABS-KEY ( fundus AND photography) OR TITLE-ABS-KEY ( fundus AND photograph) OR TITLE-ABS-KEY ( fundus AND imaging) OR TITLE-ABS-KEY ( retinal AND camera) OR TITLE-ABS-KEY ( retinal AND photography) OR TITLE-ABS-KEY ( retinal AND photograph) OR TITLE-ABS-KEY ( retinal AND imaging))) AND ( ( ( TITLE-ABS-KEY ( malaria) AND TITLE-ABS-KEY ( retina))) OR ( ( TITLE-ABS-KEY ( malarial AND retinopathy) OR TITLE-ABS-KEY ( cerebral AND malaria)))).

### Web of Science Search Strategy

(ALL = (optical coherence tomography) OR ALL = (fluorescein angiography) OR ALL = (indocyanine green) OR ALL = (photography) OR ALL = (optical imaging) OR ALL = (retinal imaging) OR ALL = (retinal camera) OR ALL = (retinal photograph) OR ALL = (retinal photography) OR ALL = (fundus imaging) OR ALL = (fundus camera) OR ALL = (fundus photograph) OR ALL = (fundus photography)) AND ((ALL = (retina) AND ALL = (malaria)) OR ALL = (cerebral malaria) OR ALL = (malarial retinopathy)).

## Appendix 2

**Table:**

Study author	Year
Retinal photography	
Soliz [23]	2016
Sayeed [21]	2011
Maude [20]	2009
Hero [8]	1997
Davis [25]	1991
Fluorescein angiography	
MacCormick [28]	2022
Barrera [22]	2018
MacCormick [27]	2015
Beare [26]	2008
Hero [8]	1997
Davis [25]	1992
Optical coherence tomography	
Tu [29]	2021
Paquet-Durand [30]	2019
Other	
Remer [38]	2018
Liu [37]	2012
Beare [35]	2006
Automated detection	
Li [48]	2022
Kurup [34]	2020
Chen [49]	2020
Yan [50]	2019
Zhao [51]	2018
Rochim [52]	2018
Joshi [53]	2018
Zhao [54]	2017
Zhao [55]	2017
MacCormick [56]	2017
Joshi [57]	2017
Akram [58]	2017
Zhao [59]	2015
Zhao [60]	2015
Joshi [61]	2015
Ashraf [62]	2015
Ashraf [62]	2015
Agurto [63]	2015
Zheng [64]	2014
Saleem [65]	2014
Joshi [66]	2012

## Abbreviations

AI: Artificial intelligence
AUC: Area under the curve
BBB: Blood brain barrier
BIO: Binocular indirect ophthalmoscopy
BRB: Blood retinal barrier
CM: Cerebral malaria
CNP: Capillary non-perfusion
DC: Dice coefficient
EPCR: Endothelial protein C receptor
FA: Fluorescein angiography
FL: Fluorescein leak
FP: False positive
FN: False negative
HRA: Hyperreflective areas
HRP-2: Histidine-rich protein 2
ICAM-1: Intercellular adhesion molecule 1
IVFD: Intravascular filling defect
LFL: Large focal leak
LMIC: Low- and middle-income countries
MR: Malarial retinopathy
OCT: Optical coherence tomography
PfEMP-1: *P. falciparum* erythrocyte membrane protein 1
PL: Punctate leak
pRBCs: Parasitized red blood cells
PST: Phase stretch transform
RNFL: Retinal nerve fibre layer
ROC: Receiver operating curve
SD-OCT: Spectral domain optical coherence tomography
SM: Severe malaria
TCV: Traditional computer vision
VL: Vessel leak
TP: True positive
TN: True negative
UM: Uncomplicated malaria
λ: Wavelength
